# True abscopal effect in a patient with metastatic non-small cell lung cancer

**DOI:** 10.1186/s13014-021-01920-4

**Published:** 2021-10-02

**Authors:** Oliver Vilinovszki, Nicolaus Andratschke, Martin Huellner, Alessandra Curioni-Fontecedro, Stephanie G. C. Kroeze

**Affiliations:** 1 Department of Medical Oncology and Hematology, University Hospital Zurich, Zurich, Switzerland; 2 Department of Radiation Oncology, University Hospital Zurich, Zurich, Switzerland; 3 Department of Nuclear Medicine, University Hospital Zurich, Zurich, Switzerland; 4 Faculty of Medicine, University of Zurich, Zurich, Switzerland

**Keywords:** Abscopal effect, Non-small cell lung cancer, Lymph node metastases, Bone metastases, Localized treatment, Systemic treatment, Radiotherapy, Immunotherapy

## Abstract

**Background:**

Systemic response to local anticancer treatment is a phenomenon called ‘abscopal effect’. The immune system is thought to play a pivotal role in its occurrence. To date, several cases have been reported, particularly in patients receiving combined local treatment and immune checkpoint inhibitors. In such cases, it is impossible to discriminate between the effects of local and systemic treatment. Only a few cases of abscopal effect have been described with radiotherapy alone.

**Case presentation:**

Here, we report on the case of an 81-year-old woman with recurrent metastatic squamous cell carcinoma of the lung with mediastinal tumor bulk, lymph node and bone metastases. The patient refused to undergo systemic treatment, and palliative stereotactic radiotherapy of the mediastinal tumor was performed. At restaging with FDG-PET/CT, the patient presented with a decrease in size and FDG-avidity both of the irradiated site and of the lymph node and bone metastases (which did not receive radiotherapy). At 25 months after radiotherapy, the patient is still in remission at all sites.

**Conclusions:**

This is a rare case of an abscopal effect after radiotherapy as monotherapy. It is one of the few hitherto reported for lung cancer. Several ongoing studies with a combination of radiotherapy and immunotherapy are seeking to exploit a potential synergy to induce abscopal effects.

## Background

The term ‘abscopal effect’ refers to the observation of a systemic effect on metastases after local intervention to one of the lesions [1]. Although radiotherapy is a local treatment, it is capable of inducing abscopal effect, as demonstrated by a limited number of cases [2–4]. Evidence from studies on the effect of radiotherapy on cancer immune response suggests the following mechanism for the abscopal effect: tumor cell destruction by irradiation promotes tumor antigen release and their presentation by antigen presenting cells; this in turn leads to an activation and expansion of antitumor lymphocytes that recognize and eliminate tumor cells even in distant metastases [5]. The elaborate work by Demaria and colleagues provided preclinical evidence. They treated mice bearing syngeneic bilateral mamma carcinoma with Flt3-Ligand to enhance dendritic cell expansion and unilateral irradiation, which lead to abscopal effect in wild-type animals but not in T-cell-deficient mice [6].

Given the role of the immune system in abscopal effects and the stimulation of antitumor immune response during immunotherapy, the recent increase of reported cases of abscopal effects is not surprising [3, 7, 8]. Yet, in many cases with immune checkpoint inhibition, no clear distinction between abscopal effect versus sole effect of immunotherapy and concurrent irradiation can be made. Even if immunotherapy had been halted before initiation of radiotherapy, delayed response to immunotherapy after pseudoprogression [9] may also be a possible explanation.

Cases of abscopal effects occurring after sole radiotherapy remain extremely rare [2–4, 7]. Here, we describe a seldom case of abscopal effect without concurrent or past systemic cancer treatment, which represents, to the best of our knowledge, the 5th case in patients with lung cancer ever reported [10–13].

## Case presentation

**Fig. 1:**
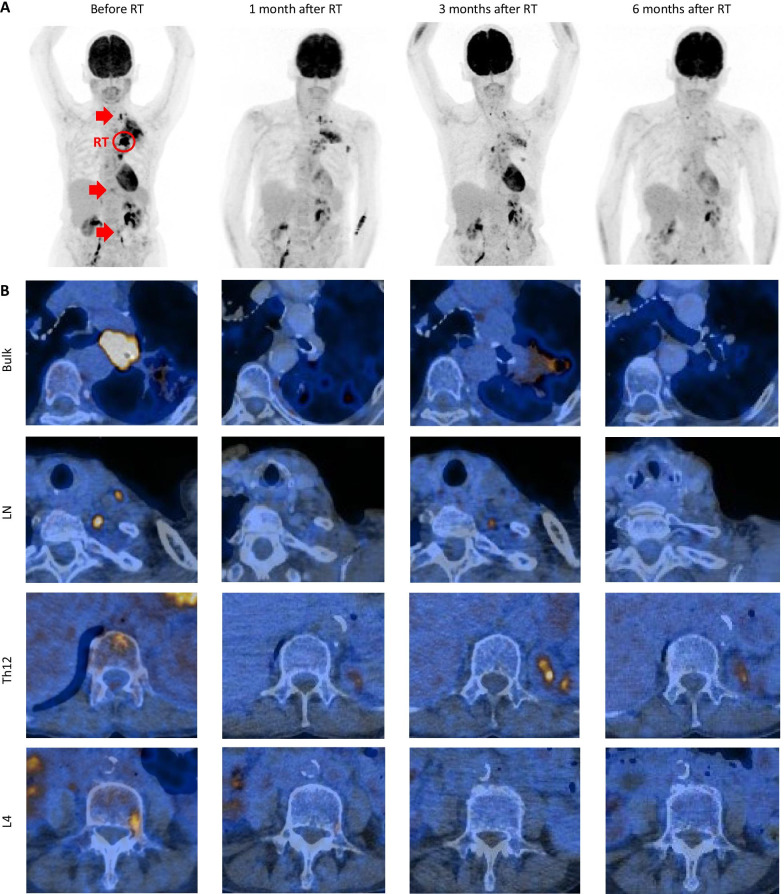
^18^F-FDG PET/CT images before irradiation and at 1, 3, and 6 months after completion of palliative radiotherapy (RT). **a** PET MIP image and **b** fused axial PET/CT images showing the initial stenotic pulmonary tumor bulk (red circle), the periclavicular lymph node (LN) metastases, the bone metastases in vertebrae Th12 and L4 (red arrows), and their regression over time. Further follow-up at 9, 12, and 19 months after radiotherapy showed persistent remission

We report on an 81-year-old woman with no relevant comorbidities and a smoking history of 5 pack years. In 2006 she was diagnosed with a pT2a, pN0 (0/5), cM0, UICC stage IB squamous cell carcinoma of the left upper lung lobe, for which she underwent a double-sleeve lobectomy with lymphadenectomy. During 5 years of follow-up, no relapse occurred. In April 2019, the patient presented with a post-stenotic pneumonia. Computed tomography of the chest showed a mass in the left lung. Radiological staging with whole-body ^18^F-FDG-PET/CT and brain MRI showed metastatic disease with a strongly metabolically active primary tumor (SUV_max_ 16.7), pleural carcinomatosis on the left side (SUV_max_ 9.4), periclavicular lymph node metastases on the left side (SUV_max_ 9.9), and bone metastases in the 12th thoracic and 4th lumbar vertebra (SUV_max_ 5.0 and 4.6, respectively) (Fig. 1). Histology of bronchoscopic biopsy specimens revealed squamous cell carcinoma, which could be interpreted as recurrence or as a second lung cancer, taking into consideration the 13-year-long disease-free interval. The immunohistochemistry for programmed cell death ligand 1 (PD-L1) revealed an expression in 20% of tumor cells and in < 10% of immune cells. In order to complete our internal lung cancer diagnostic algorithm (Treichler G, manuscript in preparation), we performed next-generation sequencing using the FDA-approved FoundationOne^®^CDx assay. We found a structural aberration on the long arm of chromosome 3 (3q) with amplifications of the genes *PIK3CA*, *SOX2*, and *FGF12*; mutations of *TP53*, *ATRX1*, *RB1*, and, subclonally, *PIK3CA*; a stable microsatellite state (MSS), and a low tumor mutational burden (5 Muts/Mb).

**Fig. 2:**
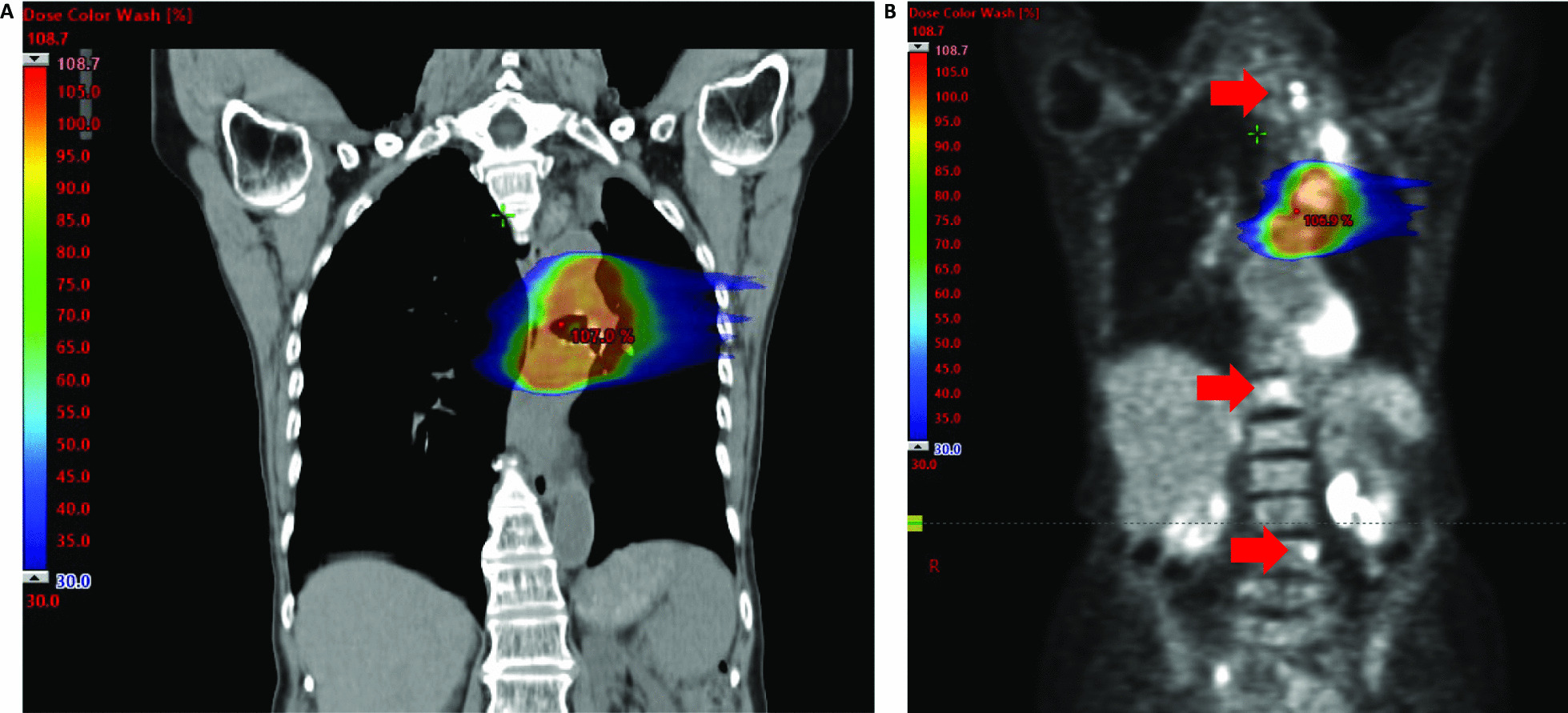
Color wash dose distribution of the applied fractionated radiotherapy with 12 × 3 = 36 Gy. Radiotherapy treatment plan merged with **a** CT and **b** PET images in sagittal plane. Color wash is shown as of 30% of the prescribed dose. FDG-positive metastases are marked with red arrows

Based on the recommendation by our multidisciplinary tumor board, a therapy regimen consisting of pembrolizumab, carboplatin and paclitaxel in analogy to the KEYNOTE-407 study [14] was suggested. The patient declined systemic treatment. She was thus referred for radiotherapy in order to achieve local control, due to the central localization of the tumor with associated risk of post-stenotic pneumonia and bleeding. The patient underwent conventionally fractionated radiotherapy of the principal pulmonary tumor bulk with palliative intention with 3 Gy in 12 fractions, resulting in a total dose of 36 Gy, using Volumetric Modulated Arc Therapy (VMAT, RapidArc™). Radiotherapy was well tolerated, with esophagitis grade 2 according to the Common Terminology Criteria for Adverse Events (CTCAE) version 5.0. Four weeks after the last day of radiotherapy, restaging was performed with ^18^F-FDG-PET/CT. A partial remission of the tumor bulk, the nodal metastases and, surprisingly, also of the vertebral lesions was shown (Fig. 1). Of note, the supraclavicular lymph nodes and the two bone metastases in the spinal column had not been included in the irradiation field with scatter doses of 0.201 Gy, 0.093 Gy, and 0.021 Gy on the lymph nodes, vertebra Th12, and L4, respectively (Fig. 2). Furthermore, no antiresorptive medication had been administered. Due to the remission and the lack of tumor-related symptoms, systemic treatment—although no longer refused by the patient—was withheld. The patient underwent radiological follow-up, during which further decrease in tumor size and complete metabolic remission of the bone, pleural and lymph node metastases was seen (Fig. 1). Currently, 25 months after radiotherapy, the patient is still free of symptoms and has an Eastern Cooperative Oncology Group (ECOG) Performance Status of 0.

## Discussion and conclusions

We describe a rare case of an elderly woman with metastatic non-small cell lung cancer, whose distant metastases regressed after radiotherapy of the mediastinal tumor bulk, in the absence of systemic treatment. Recognizing the abscopal effect was crucial to prevent unnecessary systemic treatment in an asymptomatic patient with limited tumor burden, and thus avoid toxicities. In the few previously published cases of “true” abscopal effect in lung cancer [10–13], with the exception of Hamilton’s report with oligometastatic disease [11], remission was quickly followed by progression. This emphasizes the significance of the persisting long-term remission of 25 months observed in our case.

Multiple variables, such as histological subtype, number of metastases, timing of radiotherapy (concurrent vs. sequential), the volume of irradiation, the number of irradiated sites, the chosen fractionation regimen, and the applied dose are thought to influence the likeliness of an abscopal effect [1, 8]. For instance, based on preclinical data, the type of dose fractionation influences the level of antigen presentation by tumor cells, with the best results obtained with high-dose, low fractionated radiotherapy [15]. In contrast, our case suggests that conventional fractionation might as well efficiently trigger an antitumor immune response, in line with the radiation regimens used in previously reported clinical cases with abscopal effect [2, 3, 16].

**Fig. 3:**
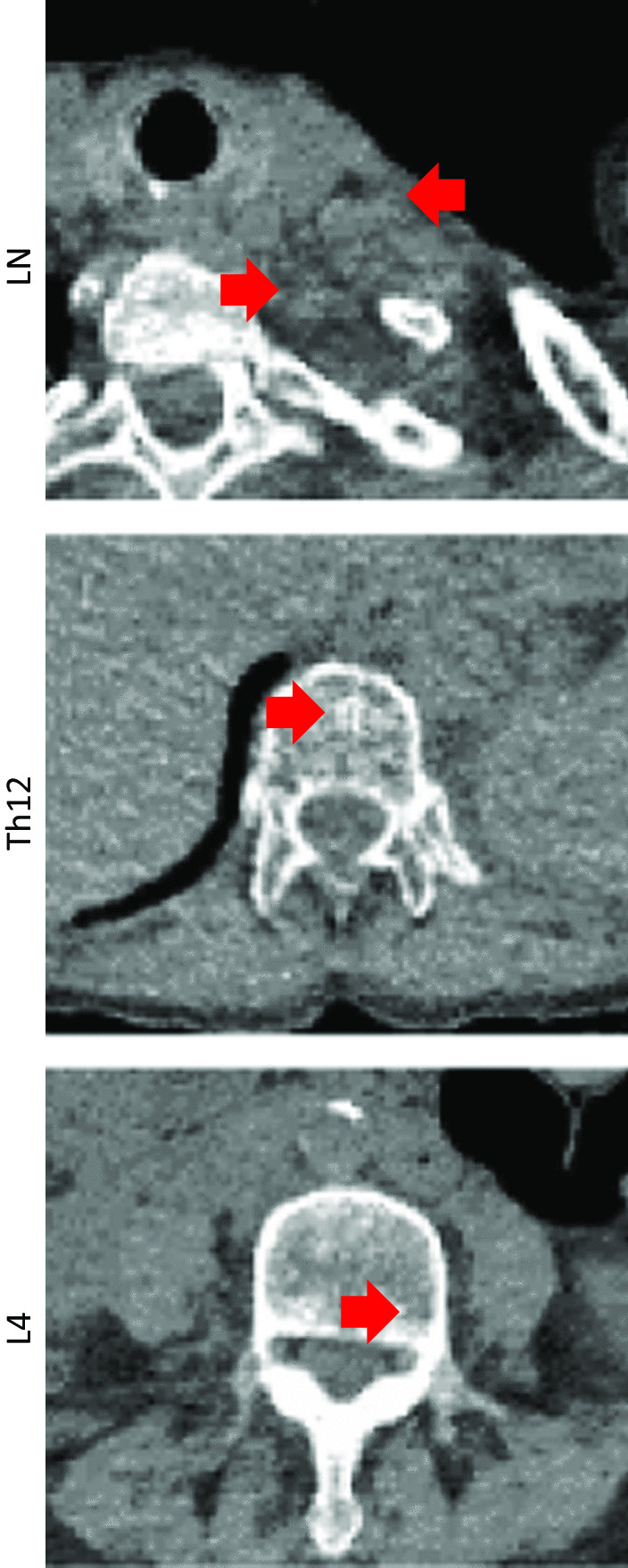
Native CT images before irradiation. The periclavicular lymph node (LN) metastases and the bone metastases in vertebrae Th12 and L4 (red arrows) are shown in axial plane

Limitations of our observation include the missing histological confirmation of the metastases. However, both the radiological evaluation and the observed simultaneous remission of the lesions make alternative diagnoses unlikely. ^18^F-FDG PET/CT imaging has an excellent specificity (98%) for the detection of bone metastases of non-small cell lung cancer [17, 18] and our patient had no other potential cause for the initially observed FDG-avidity in the vertebrae, such as a fracture, trauma, inflammation or recent cancer treatment. All metastases were also discernable on native CT images: bone lesions were sclerotic, periclavicular lymph nodes were increased in number and roundish (Fig. 3). Furthermore, the lack of histological confirmation represents a consistent limitation in the literature on abscopal effects [10–13].

The innate and adaptive immune system plays an important role in tumor control, as demonstrated by the increased incidence of cancer in immunocompromised individuals [19]. Besides systemic immunotherapy, irradiation also stimulates the immune system [5, 6]. The combination of the two strategies, i.e., radiotherapy and immunotherapy, to enhance cancer response appears therefore promising [8, 15, 20, 21]. The PEMBRO-RT study was the first major trial to show clinical efficacy in this setting, although only in the PD-L1-negative subgroup [22]. In contrast, another recently published randomized phase 2 trial in metastatic head and neck cancer patients failed to show a benefit of simultaneous stereotactic body radiation therapy [23], but results were limited by a small study population and lack of selection according to the PD-L1 status. Multiple ongoing phase I and II clinical trials are seeking to exploit such a synergistic effect further [1, 3, 8, 24, 25]. Efficacy and safety profiles of pending trials will determine the future of this novel and exciting interdisciplinary field [26].

In conclusion, we observed a rare case of a true abscopal effect after radiotherapy as monotherapy. The approach of triggering abscopal effects should be examined further in prospective clinical trials, primarily in combination with immunotherapy.

## Data Availability

The datasets used and/or analyzed during the current study are available from the corresponding author on reasonable request.

## Abbreviations

CTCAE: Common Terminology Criteria for Adverse Events
ECOG: Eastern Cooperative Oncology Group
FDA: United States Food and Drug Administration
^18^F-FDG: Fluorodeoxyglucose
L4: 4th lumbar vertebra
MIP: Maximum intensity projection
MRI: Magnetic resonance imaging
MSS: Microsatellite stable
PD-L1: Programmed cell death ligand 1
PET/CT: Positron emission tomography–computed tomography
SUV_max_: Maximum standardized uptake value
Th12: 12th thoracic vertebra
UICC: Union for International Cancer Control
VMAT: Volumetric modulated arc therapy
